# Obituary

**Published:** 1888-09

**Authors:** 


### Obituary.

DIED.—On Friday, August 24th, Dr. GEORGE W. KEELY, of Oxford, Ohio. His death was the result of an accidental fall from a window of the third story of his office building, where he was arranging some electric wires.

This announcement will be the profession a very great surprise, and a matter of sorrow to all who knew Dr. Keely. There is hardly any one in the profession who was more widely known, and who had more true and cordial friends. There will be in the next issue a more extended notice.

